# Supplementary material for: Effects of Weak Acids on the Microbiological, Nutritional and Sensory Quality of Baltic Herring (Clupea harengus membras)
Source: Foods. 2022 Jun 12;11(12):1717. doi: 10.3390/foods11121717 (PMC9222561; doi:10.3390/foods11121717)
Supplement: Supplementary file 1 [file foods-11-01717-s001.zip › foods-1727842-supplementary.pdf]

Table S1. Concentration of acids in and pH values of pickling and marinade solutions of training samples of 0 months′ sensory evaluation.

Acid	Concentration in Pickling Solution (g/L)	Concentration in Marinade Solution (g/L)	pH of Pickling Solution	pH at the End of Pickling
Acetic acid [1]	40.0	0.67	2.71	4.66
Acetic acid [2]	50.0	0.67	2.32	3.78
Citric acid [2]	20.0	0.17	1.93	3.78
Lactic acid [1]	15.6	0.17	2.12	4.00
Malic acid [2]	20.0	0.14	2.04	3.65
Tartaric acid [1]	21.5	0.14	1.65	3.30

[1] Samples used in the first week of sensory evaluation training. [2] Samples used in the second week of sensory evaluation training.
